# Delamination of Layered Double Hydroxide in Ionic Liquids under Ambient Conditions

**DOI:** 10.1021/acs.jpclett.2c03275

**Published:** 2022-12-15

**Authors:** Dóra Takács, Gábor Varga, Edit Csapó, Andrej Jamnik, Matija Tomšič, István Szilágyi

**Affiliations:** † MTA-SZTE Lendület “Momentum” Biocolloids Research Group, University of Szeged, H-6720 Szeged, Hungary; ‡ Interdisciplinary Excellence Center, Department of Physical Chemistry and Materials Science, University of Szeged, H-6720 Szeged, Hungary; § MTA-SZTE Lendület “Momentum” Noble Metal Nanostructures Research Group, University of Szeged, H-6720 Szeged, Hungary; ∥ Faculty of Chemistry and Chemical Technology, University of Ljubljana, Večna pot 113, SI-1000 Ljubljana, Slovenia

## Abstract

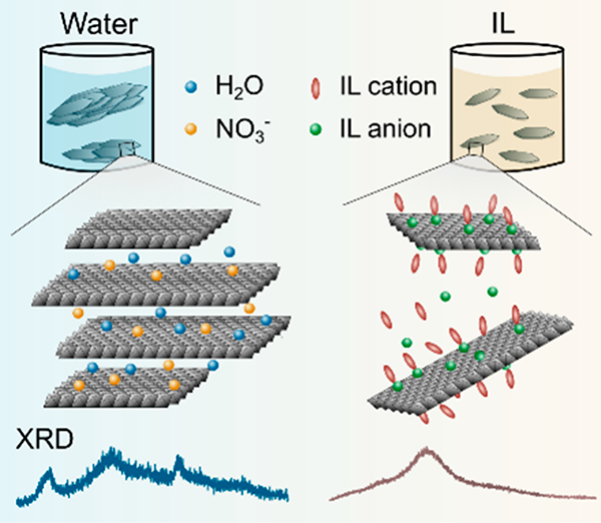

Liquid phase delamination of layered materials into single- or few-layer nanosheets leads to stable nanoscale dispersions of 2D materials. The delamination of layered double hydroxide (LDH) to double hydroxide nanosheets was studied in two ionic liquids (ILs): ethylammonium nitrate (EAN) and 1-butyl-3-methylimidazolium thiocyanate (BMIMSCN). The as-prepared lamellar structure of LDH disappeared upon dispersing it in ILs due to delamination into 2D nanosheets confirmed by X-ray scattering and diffraction techniques and further evaluated by height profile assessment of the nanoparticles by atomic force microscopy. The results showed that both the thickness and lateral size of the dispersed particles decreased in the IL-based samples, indicating that cleavage of the LDH materials can be observed in addition to delamination. The findings prove the concept of delamination of layered materials by ILs under ambient conditions—an excellent way to prepare 2D double hydroxide nanosheet dispersions in one step using nonvolatile green solvents.

The delamination of layered materials into unilamellar nanosheets has attracted considerable interest in both basic and applied disciplines due to the growing importance of such low-dimensional nanomaterials.^1−3^ These 2D nanosheets offer very interesting properties such as unique electronic, structural, and mechanical properties as well as high specific surface area.^1,4^ These properties are important for various applications such as catalysis,^5^ sensing,^6,7^ and energy storage.^8^ Although graphene is probably the most studied 2D material,^9,10^ other inorganic compounds (e.g., aluminosilicates,^11^ clays,^12,13^ metal oxides,^14^ and chalcogenides^15^) are becoming increasingly common due to their advantageous properties and the availability of the starting materials on a large scale.^16^ Among these, layered double hydroxides (LDHs) are one of the most intensively studied lamellar materials^17^ because the chemical composition of both the metal hydroxide layers and the intercalated anions can be precisely controlled, which is particularly advantageous for the desired applications.^18−20^

In general, LDHs are considered inorganic layered materials consisting of stacks of positively charged double hydroxide layers of divalent (e.g., Mg^2+^, Zn^2+^, or Ca^2+^) and trivalent (e.g., Al^3+^, Fe^3+^, or Cr^3+^) metal ions, with hydrated anions among the layers to compensate for the charge (Scheme 1). Because of their unique anisotropic structure, they are one of the few layered materials with a positive structural charge that enables the adsorption of negatively charged substances, such as biomolecules in delivery processes.^21−23^ Besides, LDHs are also used as basic building blocks for functional materials used as catalysts^5,24^ and electrodes.^25^

**Scheme 1:**
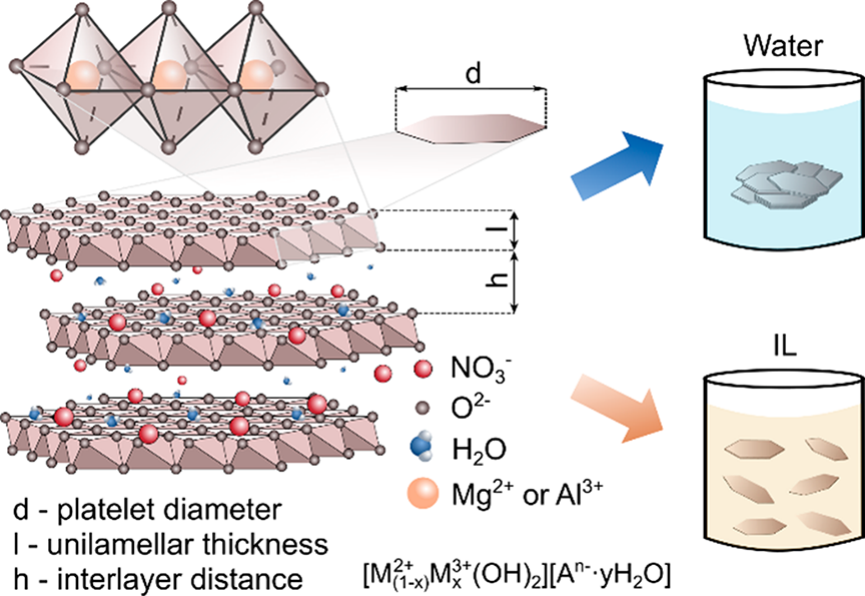
Schematic Representation of the Structure of LDHs

The most practical way to obtain nanosheets from lamellar materials is to delaminate them in the liquid phase.^1^ In this process, the layered material is dispersed in a suitable solvent, often organic, and delamination occurs after intercalation of solvent^26^ molecules or ionic species^27^ which increases the distance between the layers by weakening interlayer adhesion and thus reducing the energy barrier to delamination. However, the conditions and the extent of swelling depend on many factors, such as layer charge density and gallery ion identity.^28^ Moreover, delamination in water is difficult to perform and always requires pretreatment of the precursor lamellar LDH^29,30^ because the exfoliation is severely inhibited by the high charge density and the integrated hydrogen-bonding network between the layers. In these processes, the presence of an additional compound (e.g., polymer or surfactant) is often required to stabilize the resulting dispersion by electrostatic (or electrosteric) stabilization.

Considering these aspects, ionic liquids (ILs) are one of the most promising solvent candidates for this task.^31^ ILs are molten salts consisting entirely of ions^32−34^ and possess several advantageous properties compared to conventional solvents. These include high chemical and thermal stability, a broad electrochemical window, and low vapor pressure, to name a few. Most importantly, fundamental studies have shown that ILs can reduce the strength of attractive interactions between charged surfaces due to their interfacial assembly^35,36^ or partial dissociation,^37^ leading to the formation of stable particle dispersions in ILs.^38,39^ Ion pair formation in ILs is an important phenomenon regarding the stabilization. In dilute aqueous solutions, ILs tend to dissociate, while the ions associate and form ion pairs as the concentration increases.^36,40,41^ In this way, the shielding of attractive interactions between layers due to the repulsive solvation forces generated by the self-assembly of IL constituents on the surface should also promote the collapse of stacked structures and subsequent delamination into unilamellar nanosheets. Such a phenomenon was first demonstrated in IL–graphene systems.^42^ Among ILs, the ones with surface energies matched to the graphite substrate proved to be more effective, as they could exfoliate graphite into 2D graphene under ambient conditions, without requiring any form of external energy input.^42^ Moreover, delaminated graphene nanosheets were stabilized in IL dispersions solely by choosing the appropriate composition of ILs.^43,44^

For these reasons, ILs are very promising as delamination media, as they allow both one-step delamination in liquid phase and stabilization of the resulting 2D nanosheets in dispersions. Although composites of ILs and LDHs have been reported in the past,^45−48^ no comprehensive studies have been conducted to evaluate the potential delamination of single-phase layered LDHs into 2D double hydroxide materials with a thickness of one or a few nanosheets. Therefore, this Letter discusses the delamination of mesoporous LDHs under ambient conditions with minimal external energy input in ILs (ethylammonium nitrate (EAN) and 1-butyl-3-methylimidazolium thiocyanate (BMIMSCN)) based on systematic characterization of the as-prepared and delaminated LDH samples by X-ray scattering and diffraction as well as by microscopy. These ILs have been widely studied, and their advantageous interfacial properties as well as moderate viscosities were expected to be beneficial in delamination processes.

To check the formation of the layers, i.e., to prove the successful synthesis of the mesoporous LDH powder, X-ray diffraction (XRD), small-angle X-ray scattering (SAXS), and small- and wide-angle X-ray scattering (SWAXS) measurements were performed. The results are shown in Figure 1. The very broad XRD peaks seen in Figure 1a in the case of the dispersions (in the 2θ range from 10° to 30°) represent the (background) contributions due to the structure of the solvent used,^49^ i.e., water, EAN, and BMIMSCN. The XRD result for the LDH powder sample is also shown as a black curve in Figure 1a and shows the characteristic sharp diffraction patterns of LDH-based crystalline materials.^50^ The average crystallite size was calculated from the half-width of the (003) diffraction pattern using the Scherrer equation^50^ and estimated to be 15.4 nm.

**Figure 1:**
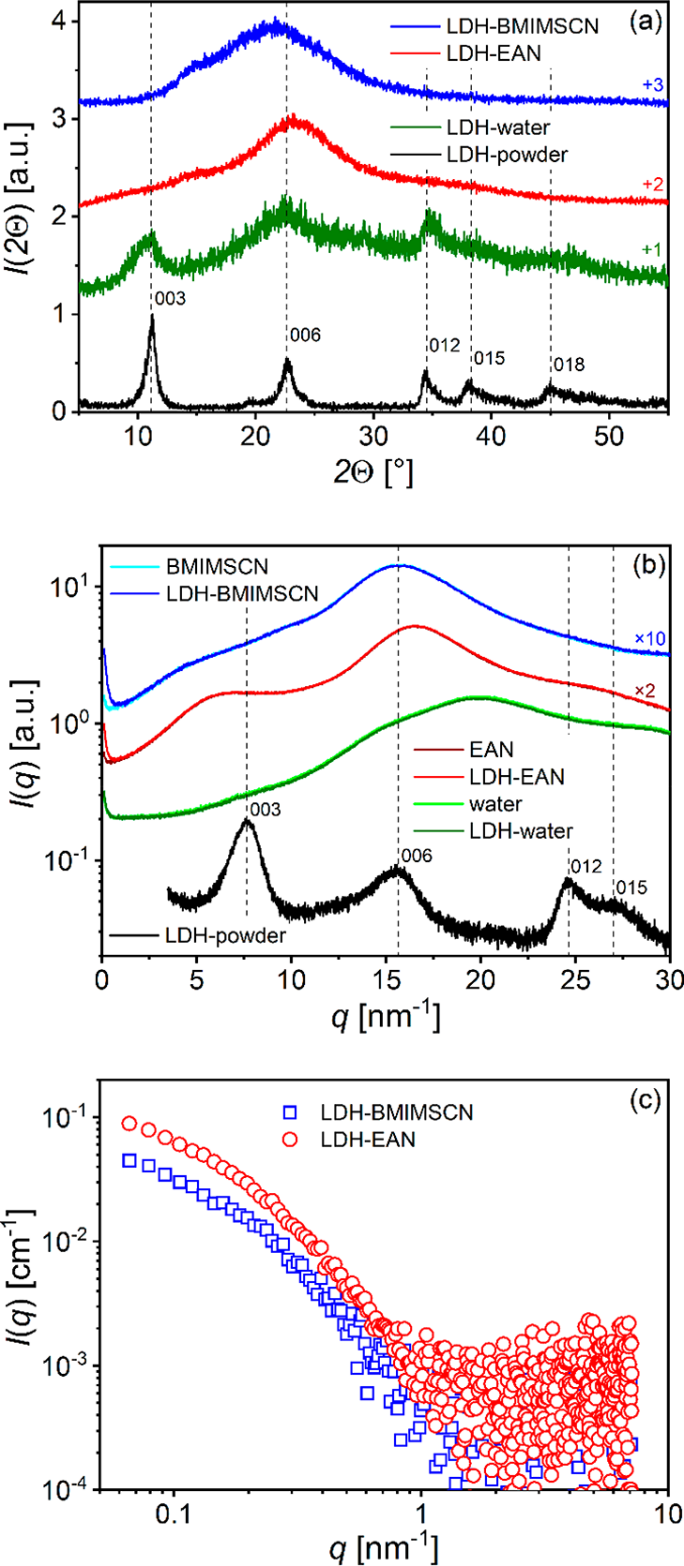
(a) Experimental XRD curves of LDH powder (black line), colloidal dispersions of LDH in water (blue line), EAN (brown line), and BMIMSCN (green line) normalized to the maximum intensity value and shifted upward in the above order for better visibility. The broad (background) peaks observed in the case of dispersions represent the scattering due to the structure of the solvent medium used, i.e., water, EAN, and BMIMSCN, respectively. (b) Raw experimental SWAXS curves of LDH samples in BMIMSCN, EAN, and water compared to the pure solvent curves and the SWAXS curve of the LDH powder sample in arbitrary units on a log-normal scale. (c) Experimental SAXS curves of LDH dispersions in BMIMSCN and EAN on a log–log absolute scale—the background solvent scattering is subtracted.

Considering the LDH basal interlayer spacing of about 0.8 nm,^51^ it is estimated that the LDH particles in this solid sample contain about 19 stacked layers. Comparing the powder diffractogram (black curve) with the sharp peaks (003, 006, 012, 015, and 018)—characteristic of the layered LDH powder structure—with the diffractogram of the aqueous LDH dispersion (green curve) in Figure 1a, it can be demonstrated that very similar diffraction patterns are also present in the latter. This indicates that the main crystalline phase of the LDH material remains unaltered in the aqueous dispersion and closely resembles the typical layered structure observed in the dry powder sample. However, the XRD results for LDH dispersions in the ILs BMIMSCN and EAN (blue and red curves, respectively) do not show such sharp peaks. The absence of these diffractions in the XRD data is a strong indication that LDH delamination has occurred in these samples under ambient conditions with minimal external energy input.

To obtain direct evidence for the presence of delaminated LDH nanosheets in these IL dispersions, SWAXS and SAXS techniques were used. The resulting experimental SWAXS and SAXS data are shown in Figure 1b on an arbitrary scale, in Figure 1c on an absolute scale, and in Figure S1 of the Supporting Information. The black curve in Figure 1b represents the SWAXS result for the LDH powder sample and shows the characteristic LDH patterns 003, 006, 012, and 015. These patterns are slightly broader than those in the XRD data of the same sample shown in Figure 1a (black curve) because the SWAXS instrument uses the line-collimated primary beam, which experimentally smears the data (and effectively broadens the scattering peaks somewhat).^52^ To clarify the scattering contribution of the pure solvent used to prepare the LDH dispersions, the raw SWAXS data for the liquid samples are shown in pairs in Figure 1b—both the scattering data of the LDH dispersion (blue, red, and dark green curves) and the pure solvent (cyan, dark red, and green curves) are presented in pairs together. Notably, the scattering curves of the two paired aqueous samples are practically identical and coincide (dark green and green curves). This means that no LDH material (particles) was detected in the aqueous dispersion. At first glance, this may seem surprising given the XRD result for the aqueous LDH dispersion in Figure 1a (dark green curve), but it should be understood that the SWAXS measurements are performed on an as-prepared, low-viscosity liquid sample, in which the crystalline LDH particles settle rapidly at the bottom of the horizontal cylindrical measurement capillary and consequently are not present in the scattering volume, or rather in the primary X-ray beam, shining through the central part of the capillary. In contrast, the liquid samples are concentrated prior to the XRD measurements to obtain a viscous, gel-like sample, in which the crystalline LDH particles cannot sediment and are still present in the primary X-ray beam of the XRD instrument. Accordingly, the SWAXS data also show the absence of LDH delamination in aqueous dispersion. They also prove the delamination of LDH crystallites in the ILs EAN and BMIMSCN and the appearance of stable LDH nanosheet liquid dispersions in these ILs.

Besides, the course of the pairwise scattering curves for the two IL samples in Figure 1b (blue and cyan curves for the BMIMSCN series; red and dark red curves for the EAN series) is practically the same over the whole range of the scattering vector except for the very low values in the SAXS region (below 2 nm^–1^), which proves the presence of stable nanoparticles in these two dispersions. Moreover, no excessive scattering is observed in the SWAXS data of the LDH liquid dispersion in Figure 1b at the positions of the scattering peaks of the crystalline LDH powder sample (peaks 003, 006, 012, and 015 of the black curve). Furthermore, the presence of the delaminated LDH nanosheets in IL dispersions can be demonstrated by the broad scattering peaks in the SAXS data in Figure 1c, where the solvent scattering was subtracted.

To support these results, an inverse Fourier transform^53,54^ (IFT) approach was used to analyze the SAXS data shown in Figure 1c. The IFT fits to the SAXS data are presented in Figures S2a and S2b. The resulting pair distance distribution functions *p*(*r*) and the thickness pair distance distribution functions *p*_t_(*r*) are shown for the delaminated LDH IL dispersions in Figures 2a and 2b, respectively. The former functions are related to the total nanoparticle size and show that the effective total nanoparticle size, which refers to the effective lateral dimensions of the delaminated LDH nanosheets, is about 60 nm in both dispersions (BMIMSCN and EAN).

**Figure 2:**
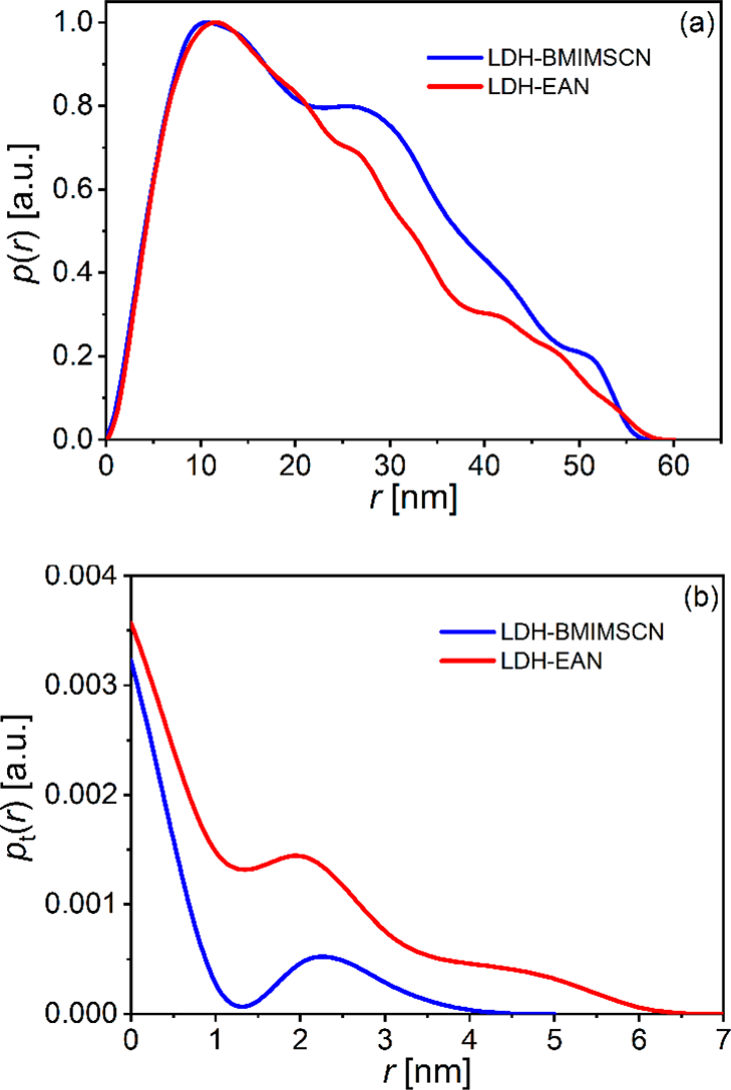
(a) Pair distance distribution function *p*(*r*) normalized to the maximal value of 1 and (b) the thickness pair distance distribution function *p*_t_(*r*) obtained by the IFT procedure from the SAXS data of LDH dispersions in BMIMSCN and EAN.

The asymmetric course of these *p*(*r*) function curves also indicates that these particles are not spherical, which is not surprising since they are assumed to be 2D nanosheets. For plate-like (lamellar) particles that are large in two dimensions, it is possible to perform the special IFT analysis mode that uses the cutoff at low values of *q* to obtain the *p*_t_(*r*) function. This function is related to the thickness of the scattering nanosheets. The curves of the *p*_t_(*r*) function shown in Figure 2b show a steep descent to a thickness of about 1.5 nm with some side wings extending to a thickness of about 4 and 6 nm. This confirms that sonication-assisted liquid delamination of the LDHs in these IL dispersions was indeed successful.

To further investigate the population of these nanosheets and possibly obtain information about their polydispersity, the atomic force microscopy (AFM) technique was used, which provided the visual results shown in Figure 3.

**Figure 3:**
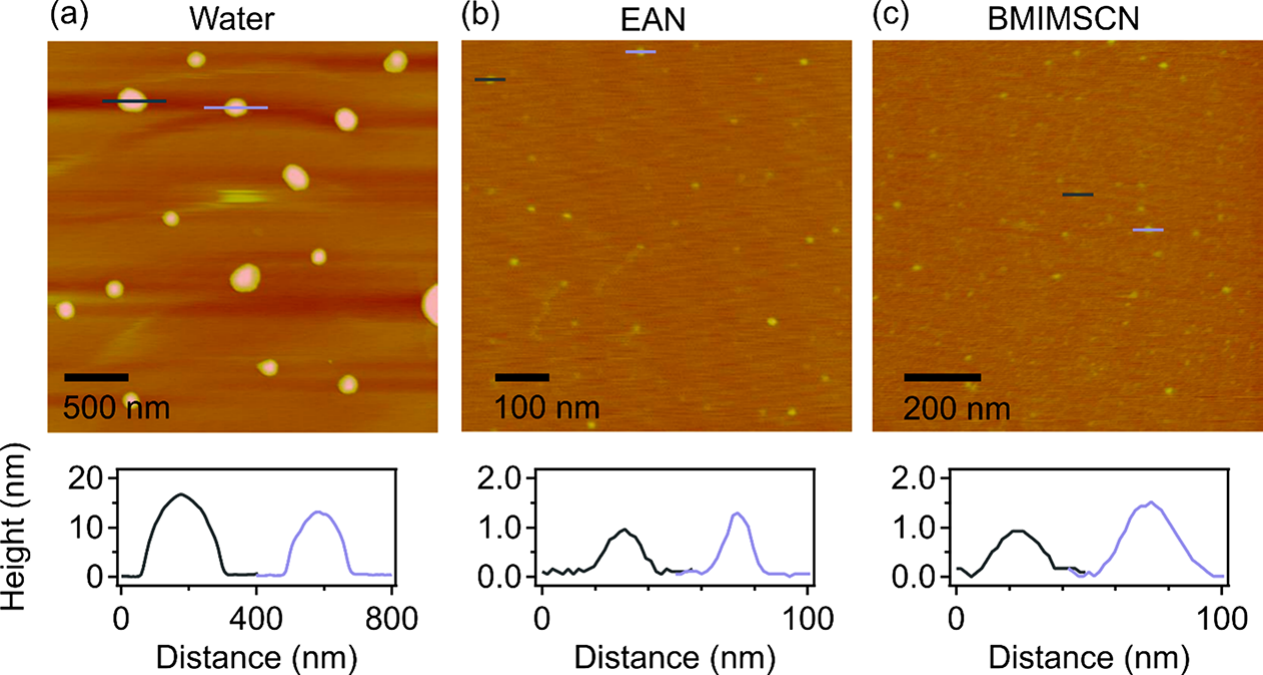
AFM images of LDHs with the associated height profiles obtained from (a) water, (b) EAN, and (c) BMIMSCN based dispersions. The presented height profiles correspond to the labeled particles.

In the aqueous LDH dispersion, the crystalline LDH particles retained their platelet-like shape and layered structure, as also shown by the AFM height profile in Figure 3a and the images in Figure S3a and S4a. Namely, large platelets with lateral dimensions of up to 400 nm can be seen. Analysis of their height profile shows thicknesses of about 15–20 nm, which is in good agreement with the thickness of about 15.4 nm determined from the XRD data (Figure 1a) for the crystalline powder sample. This indicates once more that no delamination of the LDH crystallites occurred in the aqueous dispersion.

The morphology and thickness of the dispersed LDH nanosheets as revealed by the AFM results are shown in Figures 3b and 3c (also presented in Figures S3b and S3c). The height/distance profiles in Figures 3b and 3c indicate that the thickness and lateral dimension of the LDH nanosheets in the ILs dispersions are significantly smaller compared to the crystalline platelets observed in water (see Figure 3a). The distribution histograms based on several AFM images, shown in Figure 4c–f as well as Figures S4b and S4c, also demonstrate that the average thickness of the particles decreased from 12 ± 6 nm in water to 2 ± 1 and 1.7 ± 0.8 nm for EAN and BMIMSCN, respectively, while the average lateral size of crystalline LDH platelets decreases from 187 ± 50 nm in water to the lateral size of nanosheets 32 ± 8 nm in EAN and 39 ± 8 nm in BMIMSCN. The nanosheets with lateral dimensions up to 60 nm in Figures 4d and 4f consist of up to 6 or 7 layers (but the majority of 2 or 3 layers) in Figures 4c and 4e; the perfect agreement of these findings with the SAXS and AFM results discussed above is unambiguous.

**Figure 4:**
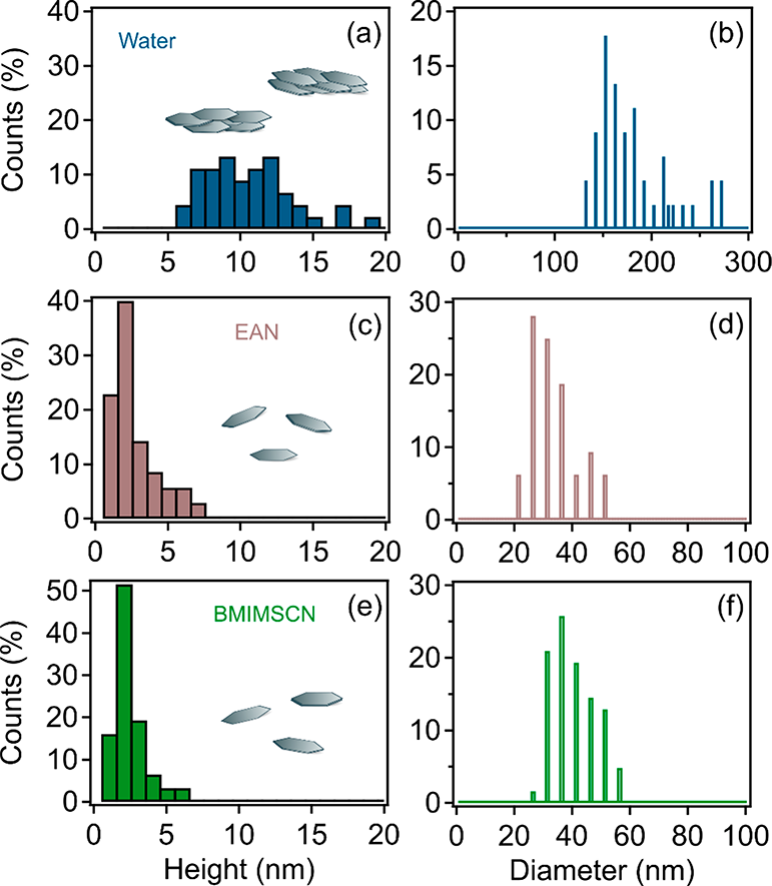
Distribution histograms of the thickness (left) and the diameter (right) after sonication-assisted solvent delamination in (a, b) water, (c, d) EAN, and (e, f) BMIMSCN.

On the basis of the results presented, one can assume either that the LDH crystallites do not change significantly when the LDH powder is dispersed in water or that the large crystalline lamellar aggregates form in aqueous LDH dispersion. However, in ILs, simultaneous disaggregation and delamination of LDH (accelerated by mild ultrasonication) occurred (Scheme 1) due to the reduced attraction between LDH sheets—similar to previous reports on IL-assisted delamination of graphene.^42^ It was assumed that IL constituents assemble on the surface of LDH in an ordered form consisting of cation and anion layers. Such an assembly of ILs on surfaces was confirmed earlier by AFM^38^ and high-energy X-ray reflectivity.^36^ Besides, direct force measurements revealed that the formation of IL interfacial layers leads to the development of repulsive oscillatory forces,^55^ which overcome attractive van der Waals and electrostatic interactions acting, in our case, between the LDH layers. Therefore, delamination occurs once oscillatory forces arise due to IL assembly on the LDH surface. It is also worth mentioning that partial delamination and cleavage of aggregates were previously reported for LDH dispersed in an organic solvent.^56^ The dispersions obtained in ILs were stable for at least 6 months, indicating a good stabilizing effect of ILs for LDH nanosheets.

In conclusion, using XRD, SWAXS, SAXS, and AFM methods, it has been shown that mesoporous LDHs can be successfully delaminated in a single step into ILs such as EAN and BMIMSCN. The disappearance of the characteristic diffraction peaks observed for crystalline LDH powder and its aqueous dispersion when LDH crystallites were dispersed in ILs confirmed the delamination of crystalline LDHs into doubly hydroxide nanosheets. This was also confirmed by SWAXS and SAXS results. The degree of delamination was further studied by AFM determining the distributions of nanosheet thickness and lateral dimensions. The latter two were compared with similar distributions obtained for aqueous LDH samples proving much smaller double hydroxide nanosheets in IL dispersions and indirectly the process of simultaneous delamination and disaggregation of LDHs in ILs. The obtained 2D double hydroxide nanosheet–IL samples were stable for months without any stabilizing agents added to the samples. These results open a new way to obtain unilamellar or multilamellar nanosheets with only a few layers in liquid dispersions in one step using a green (nonvolatile) solvent.

## Experimental Methods

Experimental details including materials used for the experiments, a description of investigation methods such as AFM, XRD, SAXS, and SWAXS, and the data analysis are described in the Supporting Information on pages S2–S5.^50,52−54,57−67^ The mesoporous nitrate-containing LDH particles studied in the present work were prepared using a novel colloidal approach.^51^ The synthesis protocol can be found in the Supporting Information. Delamination procedures were performed by dispersing the mesoporous LDH in EAN or BMIMSCN followed by ultrasonication for 1 h and stirring for 48 h.

## Supporting Information Available

Experimental details, SWAXS data, SAXS data, AFM images, and statistical analysis of AFM data (PDF)
