# Supplementary material for: Delamination of Layered Double Hydroxide in Ionic Liquids under Ambient Conditions
Source: J Phys Chem Lett. 2022 Dec 15;13(51):11850–6. doi: 10.1021/acs.jpclett.2c03275 (PMC9806852; doi:10.1021/acs.jpclett.2c03275)
Supplement: Supplementary file 1 — jz2c03275_si_001.pdf [file jz2c03275_si_001.pdf]

Supporting Information (SI)

Delamination of Layered Double Hydroxide in Ionic Liquids under Ambient Conditions

Dóra Takács,[‡,§] *Gábor Varga,*[§] *Edit Csapó,*[§,†] *Andrej Jamnik,*[‖] *Matija Tomšič,**[,‖] *István Szilágyi**[,‡,§]

[‡]MTA-SZTE Lendület "Momentum" Biocolloids Research Group, University of Szeged, H-6720 Szeged, Hungary

[§]Interdisciplinary Excellence Center, Department of Physical Chemistry and Materials Science, University of Szeged, H-6720 Szeged, Hungary

†MTA-SZTE Lendület "Momentum" Noble Metal Nanostructures Research Group, University of Szeged, H-6720 Szeged, Hungary

[‖]Faculty of Chemistry and Chemical Technology, University of Ljubljana, Večna pot 113, SI-1000 Ljubljana, Slovenia

*Corresponding authors. Email: matija.tomsic@fkkt.uni-lj.si (M.T.); szistvan@chem.u-szeged.hu (I.S.)

EXPERIMENTAL AND METHODS

Materials. Magnesium(II) nitrate hexahydrate ($Mg(NO_3)_2 \times 6H_2O$), aluminum(III) nitrate nonahydrate ($Al(NO_3)_3 \times 9H_2O$), sodium dodecyl sulphate (SDS), sodium hydroxide (NaOH) stock aqueous solution, sodium nitrate ($NaNO_3$), potassium dichromate ($K_2Cr_2O_7$), 96 m/m% ethanol (EtOH), 25 m/m% ammonia aqueous solution (NH_4OH), sodium chloride (NaCl) and the ionic liquid (IL) 1-butyl-3-methylimidazolium thiocyanate (BMIMSCN) were purchased from Sigma-Aldrich, while the IL ethylammonium nitrate (EAN) was purchased from IoLiTech GmbH. All chemicals were used as received. High-purity water was produced by a Puranity TU+ system (VWR).

To prepare the mesoporous layered double hydroxide (LDH) particles, 100 mL of 30.0 mM SDS was added to the dispersion of the original LDH obtained by the co-precipitation method.[1] The resulting slurry was stirred at 60 °C and pH 8.5 for 12 hours. Then, the SDS content was removed by calcination at 510 °C for 12 hours, and the resulting layered double oxide compounds were rehydrated to reconstruct the LDH structure. After that, the obtained slurry was stirred at 50 °C for 96 hours, followed by repeated filtration, washing and drying steps to obtain the final product.

For the liquid phase exfoliation method yielding a delaminated nanosheet dispersions of LDH in ILs, 20 mg of LDH powder was dispersed in 4 mL of various IL solvents. The obtained dispersions were ultrasonicated for 1 hour and then, mixed in a vertical rotator for 2 days.

X-Ray Diffractometry. X-ray diffraction (XRD) measurements were performed on a Bruker D8 Advanced diffractometer with CuKα (α = 0.1542 nm) as a radiation source in the 5-80° 2θ range applying 0.02° step size and using a QT026 Quartz XRD sample holder (cover plate: 20×20×0.5 mm). No background subtraction was applied. Prior to XRD measurements, the liquid samples were concentrated by membrane filtration to obtain a highly viscous, gel-like material, in

which sedimentation of larger particles was significantly reduced. Based on the XRD results, the thickness of the LDH crystals (L_c) was calculated using the Scherrer equation:[2]

$$L_c = \frac{\lambda K}{\beta \text{ co}} \quad (1)$$

where λ is the X-ray wavelength, K is the shape factor (0.9 for LDHs), θ is the Bragg diffraction angle and β is the full width at half maximum of the (003) reflection.

Small-angle X-ray Scattering (SAXS) and Small- and Wide-angle X-ray Scattering (SWAXS). SAXS and SWAXS measurements were performed with two different laboratory-modified old-Kratky type cameras (Anton Paar) connected to a conventional X-ray generator (GE Inspection Technologies, SEIFERT ISO-DEBYEFLEX 3003). The generator contained a sealed X-ray tube with a Cu anode operating at 40 kV and 50 mA. Focusing multilayer optics (Goebel mirror) was used to focus and monochromatize the primary X-ray beam to obtain a high-intensity Cu-K_α line with a wavelength, λ, of 1.54 Å. Passing this beam through a block-collimation unit provided a well-defined line-collimated primary beam. Samples were measured at 25 °C in a standard quartz capillary (outer diameter of 1 mm and wall thickness of 10 μm). SWAXS measurements were recorded on a 2D imaging plate irradiated with scattered X-rays for 30 min and read out with a delay of 5 min using a Fuji BAS 1800II imaging-plate reader with a spatial resolution of 50×50 μm^2/px. SWAXS data were obtained in the range of the scattering vector (q) from 0.1 to 30 nm^{-1}, while q can be calculated as:

$$q = \frac{4\pi}{\lambda} sin\left(\frac{\vartheta}{2}\right) \quad (2)$$

where ϑ is the scattering angle. They were corrected for sample X-ray absorption and background scattering and transformed to absolute scale using water as the secondary standard.[3] The resulting SWAXS data were still experimentally smeared due to the finite dimensions of the primary beam.[4]

Atomic Force Microscopy. The physical dimensions of the dispersed nanosheets were further investigated with a Multimode Nanoscope IIIa atomic force microscope (AFM, Digital Instruments) operating in tapping mode at room temperature and using a Si tip cantilever (PPP-NCHR-10, NanoSensors) with a nominal tip radius smaller than 9 nm and a resonant frequency of 204-497 kHz. Samples were prepared by placing a drop of the dispersion on a freshly cleaved mica (Ted Pella, Highest Grade V1) and allowing it to sediment for 1 hour. Then the IL solvent residue was removed by rinsing with acetonitrile and ultrapure water, followed by drying with N_2 gas. The height- and amplitude-mode images were acquired simultaneously at a scan rate of 1.0 Hz. Image processing and analysis were performed offline using Nanoscope V614r1 software.

Indirect Fourier Transformation (IFT) Method. To analyze the experimental SAXS data, the Indirect Fourier Transformation (IFT) method[5, 6] was employed using Generalized Indirect Fourier Transformation (GIFT) software package.[7-12] Since the maximum size of the scattering particles in the liquid dispersions studied was close to the upper limit of our experimental resolution, the typical IFT analysis was first performed providing the pair distance distribution function (PDDF), $p(r)$, which is related to the scattering intensity $I(q)$ via the Fourier transform:[5, 6]

$$I(q) = 4\pi \int_0^{\infty} p(r) \, \frac{sin(qr)}{qr} \, dr \qquad (3)$$

where r represents the distance in real space (the distance between two scattering centers within the scattering particle).

The $p(r)$ contains the information about the geometric properties of the scattering particles. In the case of flat scattering particles with a large lateral size with respect to their thickness, the structural information about the thickness of the lipid bilayer can be obtained from the scattering curves by applying a special cut-off-based mode of the IFT technique, which cosine-transforms the function $I(q)q^2$ into real space and provides the thickness $p_{\mathrm{t}}(r)$ as follows:[6, 13, 14]

$$I(q)q^2 = 4\pi A \int_0^\infty p_t(r)\, cos(qr)\, dr\ , \tag{4}$$

where A is the area of the basal plane. In this procedure, the scattering curves must be strongly truncated (cut-off) in the region of very small q values to exclude the part of the scattering curve that is strongly influenced by the scattering contribution resulting from the large lateral dimensions of the flat scattering particles. The resulting $p_t(r)$ function serves as a tool to determine the geometry of the scattering particles.[5, 6, 13-15]

ADDITIONAL RESULTS

In Figures S1-S4, additional experimental and data evaluation results obtained by SWAXS, SAXS and AFM are presented to support the discussion in the letter.

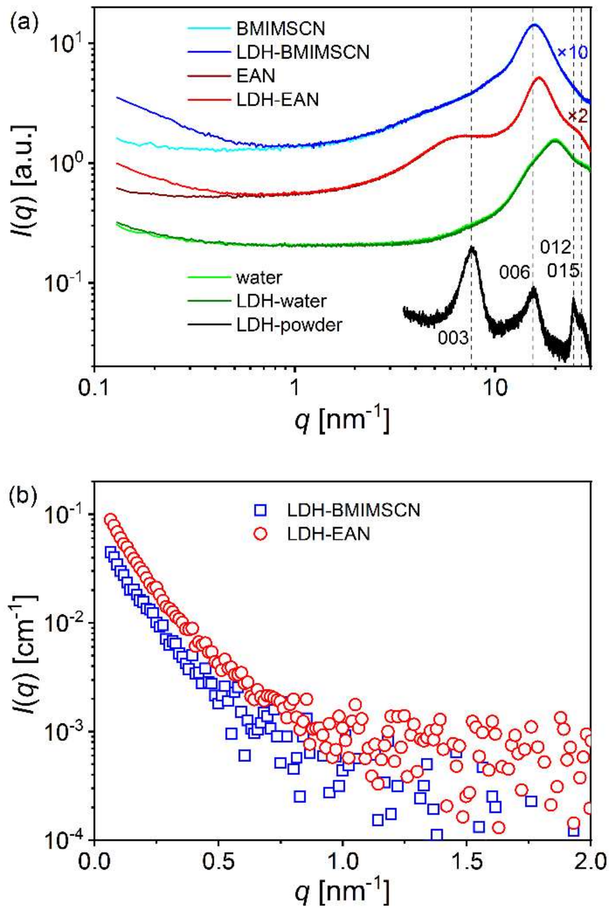

Figure S1. (a) Raw experimental SWAXS curves of LDH samples in BMIMSCN, EAN and water compared to the pure solvent curves and the SWAXS curve of LDH powder sample on arbitrary unit log-log scale. (b) Experimental SAXS curves of LDH dispersions in BMIMSCN and EAN on log-normal absolute scale. The background solvent scattering is subtracted.

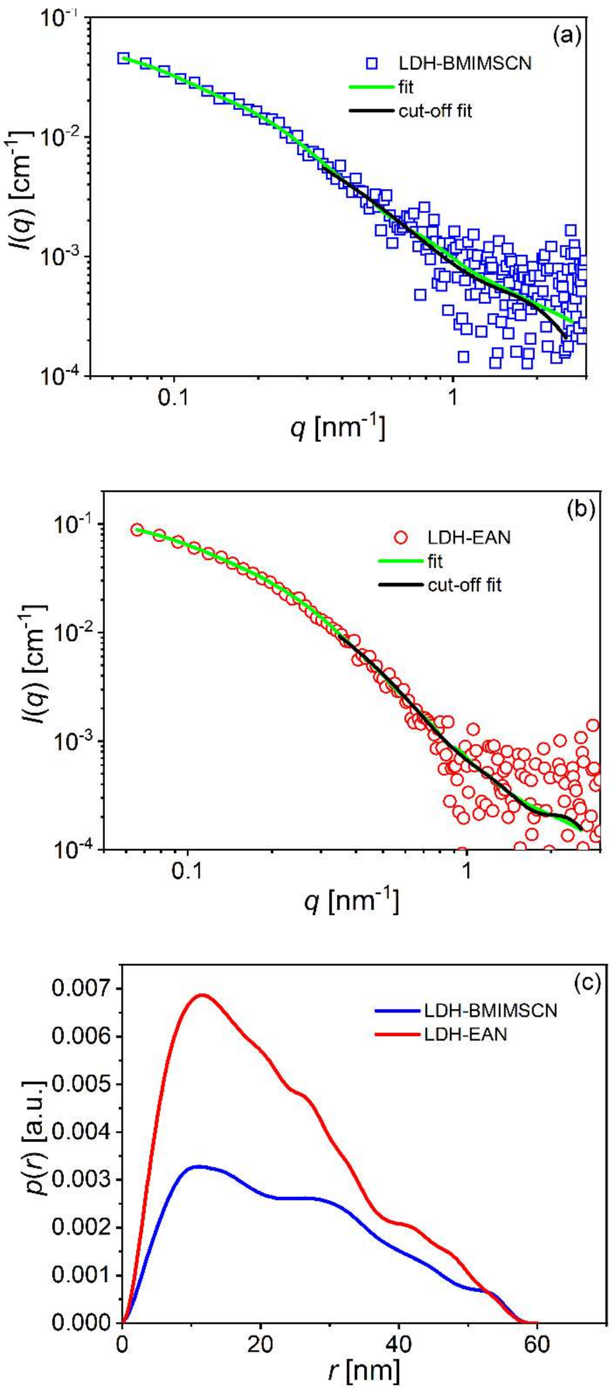

Figure S2. IFT fit and cut-off IFT fit to the experimental SAXS curves of LDH dispersions in (a) BMIMSCN and (b) EAN. (c) Resulting non-normalized $p(r)$ functions of LDH dispersions in BMIMSCN and EAN.

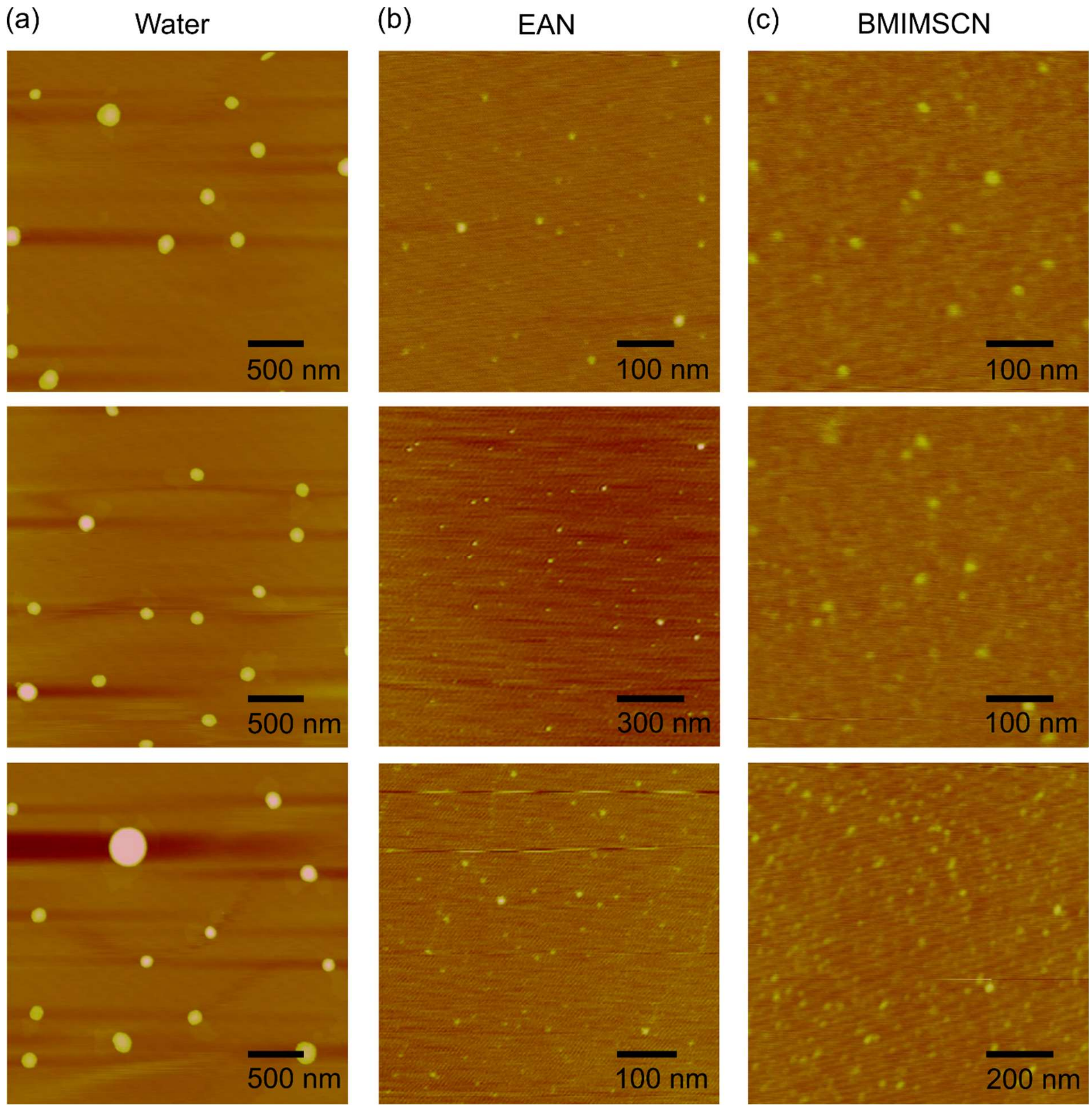

Figure S3. AFM images of the LDH particles deposited on mica substrate from water (a), EAN (b) and BMIMSCN (c).

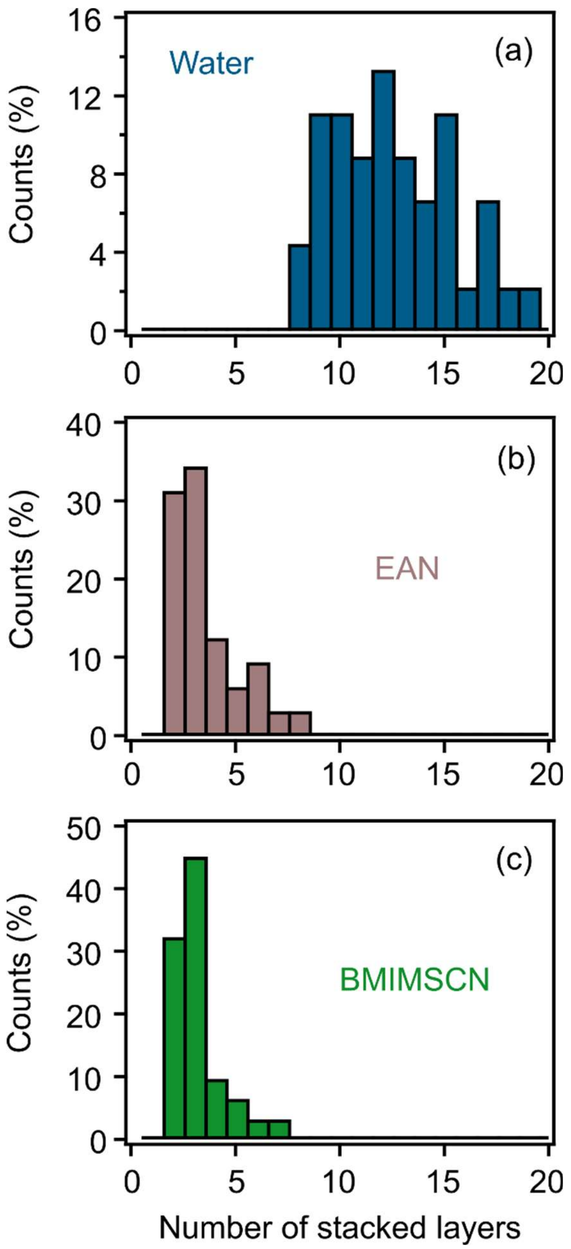

Figure S4. The distribution histograms of the stacked LDH layer numbers after dispersing the mesoporous LDH material into water (a), EAN (b) and BMIMSCN (c).

REFERENCES

1. He, J.; Wei, M.; Li, B.; Kang, Y.; Evans, D. G.; Duan, X., Preparation of layered double hydroxides. In *Layered Double Hydroxides*, Duan, X.; Evans, D. G., Eds. 2006; Vol. 119, pp 89-119.
2. Evans, D. G.; Slade, R. C. T., Structural aspects of layered double hydroxides. In *Layered Double Hydroxides*, Duan, X.; Evans, D. G., Eds. 2006; Vol. 119, pp 1-87.
3. Orthaber, D.; Bergmann, A.; Glatter, O., SAXS experiments on absolute scale with Kratky systems using water as a secondary standard. *J. Appl. Crystallogr.* **2000,** *33*, 218-225.
4. Glatter, O., Numerical methods. In *Scattering methods and their application in colloid and interface science*, Elsevier: Amsterdam, 2018; pp 137-174.
5. Glatter, O., New method for evaluation of small-angle scattering data. *J. Appl. Crystallogr.* **1977,** *10*, 415-421.
6. Glatter, O., Evaluation of small-angle scattering data from lamellar and cylindrical particles by the indirect transformation method. *J. Appl. Crystallogr.* **1980,** *13*, 577-584.
7. Brunner-Popela, J.; Mittelbach, R.; Strey, R.; Schubert, K. V.; Kaler, E. W.; Glatter, O., Small-angle scattering of interacting particles. III. D_2O-$C_{12}E_5$ mixtures and microemulsions with n-octane. *Journal of Chemical Physics* **1999,** *110* (21), 10623-10632.
8. Weyerich, B.; Brunner-Popela, J.; Glatter, O., Small-angle scattering of interacting particles. II. Generalized indirect Fourier transformation under consideration of the effective structure factor for polydisperse systems. *J. Appl. Crystallogr.* **1999,** *32*, 197-209.
9. Fritz, G.; Bergmann, A.; Glatter, O., Evaluation of small-angle scattering data of charged particles using the generalized indirect Fourier transformation technique. *Journal of Chemical Physics* **2000,** *113* (21), 9733-9740.
10. Fritz, G.; Glatter, O., Structure and interaction in dense colloidal systems: evaluation of scattering data by the generalized indirect Fourier transformation method. *J. Phys.-Condes. Matter* **2006,** *18* (36), S2403-S2419.
11. Glatter, O., Interpretation of real-space information from small-angle scattering experiments. *J. Appl. Crystallogr.* **1979,** *12*, 166-175.
12. Fruhwirth, T.; Fritz, G.; Freiberger, N.; Glatter, O., Structure and order in lamellar phases determined by small-angle scattering. *J. Appl. Crystallogr.* **2004,** *37*, 703-710.
13. Sato, T.; Sakai, H.; Sou, K.; Medebach, M.; Glatter, O.; Tsuchida, E., Static structures and dynamics of hemoglobin vesicle (HbV) developed as a transfusion alternative. *Journal of Physical Chemistry B* **2009,** *113* (24), 8418-8428.
14. Iampietro, D. J.; Brasher, L. L.; Kaler, E. W.; Stradner, A.; Glatter, O., Direct analysis of SANS and SAXS measurements of catanionic surfactant mixtures by Fourier transformation. *Journal of Physical Chemistry B* **1998,** *102* (17), 3105-3113.
15. Glatter, O., Data treatment. In *Small angle x-ray scattering*, Glatter, O.; Kratky, O., Eds. Academic Press Inc.: London, 1982; pp 119-165.
